# Comparison of the Nutritional Adequacy of Current Food-Based Very Low Energy Diets: A Review and Nutritional Analysis

**DOI:** 10.3390/nu16172993

**Published:** 2024-09-05

**Authors:** Shirley Wing Yan Poon, Robyn Mary Brown, Priya Sumithran

**Affiliations:** 1 Department of Medicine, St Vincent’s Hospital Melbourne, University of Melbourne, Fitzroy, VIC 3065, Australia; shirley.poon@unimelb.edu.au; 2 School of Agriculture, Food and Ecosystem Sciences, Faculty of Science, University of Melbourne, Parkville, VIC 3010, Australia; 3 Department of Pharmacology and Biochemistry, University of Melbourne, Melbourne, VIC 3010, Australia; 4 Department of Surgery, School of Translational Medicine, Monash University, Melbourne, VIC 3004, Australia; 5 Department of Endocrinology and Diabetes, Alfred Health, Melbourne, VIC 3004, Australia

**Keywords:** very low energy diet, VLED, nutritional adequacy, dietary fiber, micronutrient, weight loss

## Abstract

Very low energy diets (VLEDs) contain <800 kcal/day and typically comprise formulated meal replacement products with adequate protein and micronutrients. Food-based VLEDs are an alternative approach, but it is uncertain whether they can provide adequate nutrition within an 800 kcal/day restriction. This analysis aimed to assess the nutritional adequacy of food-based VLEDs compared with formula VLEDs. A systematized literature review was conducted to identify balanced food-based VLEDs by searching five scientific databases from inception to 23 March 2023 and online sources between 1 and 7 May 2023. Ultimately, nine diets were analyzed for nutritional content and compared with *Codex Alimentarius* standards for formula foods, and Australian estimated average requirement and adequate intake (AI) for adults 19–50 years. Optifast^®^ was used as a comparator. None of the VLEDs met all nutritional benchmarks. Three food-based diets had nutrient profiles similar to formula VLEDs, with one being adequate for all nutrients except thiamine, magnesium and zinc in men and iron in women. All VLEDs, including Optifast^®^, did not meet AI for dietary fiber, except one. In general, food-based VLEDs offered more fiber than Optifast^®^. In conclusion, food-based VLEDs were inadequate in certain micronutrients but offered more dietary fiber than formula VLEDs. These nutritional deficits do not preclude food-based VLEDs from being recommended, provided they are addressed.

## 1. Introduction

The rising global prevalence of obesity has increased the need for effective and accessible obesity management strategies. Among a range of interventions available, very low energy diets (VLEDs) induce rapid and substantial weight loss through severe caloric restriction to less than 800 kcal/day [1]. They are recommended for use under medical supervision in individuals with a body mass index (BMI) ≥ 30 kg/m^2^ or a BMI > 27 kg/m^2^ with at least one weight-related co-morbidity, and, in certain situations, requiring rapid weight loss, such as for reduction in liver size prior to bariatric surgery [2,3]. Contraindications of VLEDs include pregnancy, lactation, recent cardiovascular event, porphyria and severe uncontrolled psychiatric conditions [1,2]. When used as intended, VLEDs typically result in weight losses of 1.5 to 2.5 kg/week or 10–15% over a 12-week period [4]. VLEDs are typically used for 8 to 16 weeks.

VLEDs are typically implemented as total meal replacement diets (hereon called “formula VLEDs”), where all usual meals are replaced by formulated meal replacement products [4]. Specific compositional standards for formula VLEDs are outlined in the United Nation’s *Codex Alimentarius* standard CXS 203-1995 [5]. According to these standards, formula VLEDs are required to contain no less than 50 g of high-quality protein (containing all essential amino acids), no less than 50 g of carbohydrate and sufficient quantities of essential fatty acids, vitamins and minerals within an 800 kcal/day limit [5]. A summary of the nutrients and their quantities specified by the *Codex Alimentarius* standards can be found in Supplementary Materials (Table S1).

The rapid and substantial weight losses offered by VLEDs are a distinct advantage over conventional weight loss diets. Rapid weight loss can be particularly motivating for individuals with a history of unsuccessful dieting [6], which may also explain their high adherence rates [7]. A randomized trial comparing rapid weight loss using a VLED and gradual weight loss using a conventional calorie-reduced diet found that those losing weight rapidly were more likely to achieve a substantial weight loss goal (≥12.5%) compared with those losing weight gradually [6]. A formula approach may also lead to substantial weight losses and may achieve remission of early type 2 diabetes, as demonstrated in the Diabetes in Remission Clinical Trial (DiRECT) [8]. In this trial, nearly half (46%) of participants taking a total meal replacement diet of 825–853 kcal/day (slightly higher than a VLED) achieved diabetes remission (defined as having an HbA1c < 6.5% and being off all diabetes medications for at least 2 months from baseline to 1 year) compared with only 4% receiving standard care. At 12 months, mean weight losses were 10.0 kg (SD 8.0) in the meal replacement group and 1.0 kg (SD 3.7) in the standard care group, respectively [8]. Meal replacement products are also considered convenient and easily accessible [9]. As indicated by a recent systematic review of qualitative research in user experiences, formula VLEDs are well accepted, easier to adhere to than anticipated and overall viewed in a positive light by their users [7]. 

Despite these advantages, formula VLEDs have several disadvantages. Some users report struggling with managing social events and environments involving food while on a formula VLED [7]. Formula VLEDs lack adequate and varied dietary fiber. Higher dietary fiber intake has been consistently associated with multiple health benefits compared with lower dietary fiber intake, especially if sourced from whole foods in their natural state rather than synthetic or extracted forms [10,11]. These include reduced risks of cardiovascular disease, strokes, colorectal and rectal cancers, diabetes, all-cause mortality and mortality associated with coronary heart disease and cancer in epidemiological studies [10,11]. Formula VLEDs also consist almost entirely of ultra-processed food products (defined by the NOVA food classification system by Monteiro et al. as “ingredients available mostly of exclusive industrial use that result from a series of industrial processes” [12]). Although studies are inconsistent or contradictory, there is evidence suggesting that ultra-processed formula VLEDs may have negative effects on the gut microbiome, potentially reducing beneficial (butyrogenic) gut microbes while increasing potentially harmful and pathogenic gut microbes (from the Bacteroidetes phylum) [13].

An alternative to formula VLEDs would be to use conventional foods to reach the target caloric intake of ≤800 kcal/day. However, such a diet faces the challenge of being nutritionally adequate because regular foods are generally less nutrient-dense than formula VLEDs. Low-energy diets at 800–1600 kcal/day are unlikely to be nutritionally complete, as stated in the current National Institute for Health and Care Excellence (NICE) clinical guidelines for obesity [14], so further restriction to below 800 kcal/day is even more unlikely to provide adequate micronutrients.

This raises concerns about the potential for micronutrient deficiencies in food-based VLEDs, which is especially significant given that individuals with overweight or obesity are already at a higher risk of having multiple micronutrient deficiencies than lean individuals [15]. However, the evidence supporting these theoretical concerns is limited, as the nutritional adequacy of <800 kcal/day diets in adults with obesity has not been previously described. Therefore, this study aims to assess the nutritional adequacy of available food-based VLEDs in adults with obesity, and compare their nutritional composition against formula VLEDs. The results of this study will offer insights for clinicians in recommending food- or formula-based VLEDs to patients with obesity.

## 2. Materials and Methods

### 2.1. Search Strategy

A systematized literature review was used to identify balanced food-based VLEDs suitable for further nutritional analysis. Systematized literature reviews apply a uniform and comprehensive search strategy across multiple databases but do not require critical appraisal of study design, methods and outcomes [16]. Initial searches were performed in 5 scientific databases (MEDLINE (Ovid), PubMed, Cochrane Library, CINAHL and EBSCO) from their inception to 23 March 2023. As food-based VLEDs were inconsistently described across the literature, various key terms were used to encompass a variety of descriptors including “VLED”, “VLCD”, “very low energy diet”, “protein-sparing modified fast” and “semi-starvation diet”. The latter two key terms were included as their use in the literature describes different diets with varying compositions. The complete search strategy, including search terms, is presented in Supplementary Materials (Table S2). 

After performing each of the search queries, duplicates were removed, titles and abstracts screened, and full-text articles written in English were obtained and read. Reference lists were manually searched for additional articles. All article types were included, such as journal articles, review articles, books and book chapters. Search outputs were exported and managed in EndNote X9.3.3 software (Thomson Reuters, Philadelphia, PA, USA). Corresponding authors were contacted to supply a detailed menu plan of the diet used in their article.

Additional diets were then identified via an online search using the Google search engine with incognito mode between 1 and 7 May 2023 to avoid biases arising from browsing history, location and/or other personalization services. Searches were conducted using search terms including “800 kcal diet”, “800 calorie meal plan”, “fast diets” and “fast 800”. All searches and article selections were performed by one of the authors (S.P.).

### 2.2. Inclusion/Exclusion Criteria

**Figure 1:**
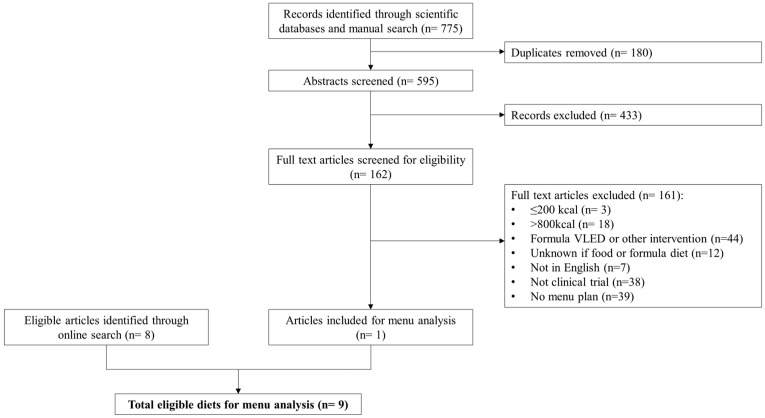
Flow chart summarizing articles and diet selection for menu analysis.

A flow diagram that summarizes article selection is presented in Figure 1. To be eligible for inclusion, diets described in the article needed to be “balanced” (i.e., include 5 core food groups described by the Australian Dietary Guidelines [17]), prescribe ≤800 kcal/day from conventional foods only and supply a detailed menu plan either as part of the original publication or provided on request to corresponding authors. Conventional foods were defined as any food or beverage item covered by the Australian New Zealand Food Standards Code, excluding Part 2.9 Special Purpose Foods [18]. Diets were excluded if they involved a commercial formula food or meal replacement component, intermittent fasting and alternate day fasting diets or where the energy content of the diet was not reported or could not be determined. Complete fasting or starvation diets (≤200 kcal/day) were also excluded, as the focus was on clinically acceptable VLEDs (i.e., potential to be recommended in clinical practice).

### 2.3. Selection of Menu Plans

Menu plans for 7 days’ duration were selected and analyzed for each of the diets by a qualified Accredited Practicing Dietitian (S.P.). Where diets provided multiple weekly menus (e.g., Week 1 to 4), the first menu plan (i.e., Week 1) was selected. Where diets provided a single-day menu plan with multiple options at each meal, 7 variations were modeled and analyzed to represent 7 days’ worth of menu plans. Food selections were chosen within guidelines of the overall diet instructions. For example, if “1 portion of fruit, e.g., 1 large banana, apple, pear, orange or grapefruit” was on the menu, then each of these fruits was entered on successive occasions where fruit was indicated on the menu plan.

### 2.4. Analysis of Menus

Sampled menus were entered into Foodworks 10 (Xyris Software, Brisbane, Australia) using the Australian Food Composition Database (AFCD Release 1, Food Standards Australia New Zealand, 2019) by a qualified Accredited Practicing Dietitian (S.P.). The AFCD database provides mostly analytical nutrient data and contains the most complete set of data for micronutrients for the Australian food supply [19]. However, data for some nutrients were missing or incomplete in this database and were not accessed from other data sources, so analyses were not conducted for pantothenic acid, vitamin D, vitamin K, choline, molybdenum, copper, chromium, manganese or fluoride. Food selections that most closely matched the item in the menu plan “as consumed” were selected (i.e., cooked, if appropriate). Where menu descriptions offered more than 1 variation on an ingredient, the 1st option was entered. Optional food items or ingredients were not entered, e.g., “season with salt/pepper/herbs to taste”.

Portion sizes were entered as described. If household measures (e.g., 1 tsp, 1 tbsp, 1 cup) were unavailable for entering, weights were estimated using the AusFood 2019 database in Foodworks 10. If portions were not specified, standard serve sizes from the Australian Guide to Healthy Eating were used. For foods that were classifiable by the core food groups in the Australian Guide to Healthy Eating, 1 standard serve was entered, e.g., spinach (vegetables food group) = 75 g or walnuts (meat, fish, poultry and alternatives food group) = 30 g. For non-specific measures (e.g., drizzle, pinch, handful), standardized amounts described in Supplementary Materials (Table S3) were used. For recipes and mixed dishes, 1 serving was entered unless otherwise specified in the menu. No dietary supplements or other sources of nutrients were entered. 

### 2.5. Assessment of Nutritional Adequacy

To determine the likelihood of nutritional adequacy, diets were compared to the Australian National Health and Medical Research Council (NHMRC)’s nutrient reference values, specifically estimated average requirements (EAR) and adequate intakes (AI) for adult men and women aged 19–50 years [20]. 

EAR is the median daily nutrient requirement for half of a healthy population [20]. Menu provisions below EAR are considered inadequate and provisions above EAR are considered adequate. Nutrients without an EAR are assigned an AI, which is the median daily nutrient intake of apparently healthy individuals and considered adequate for most people in a given population group [20]. Unlike the EAR, which relates to an observed minimum intake level required to maintain nutriture (i.e., nutrient requirement), AI is based on estimations or approximations of intake, so assessing nutritional adequacy using AI is less certain [20,21]. Interpretation of AI is limited to determining whether a diet is above or below the AI value, rather than providing a definitive assessment of adequacy. Intakes meeting or exceeding AI may be considered adequate; however, adequacy cannot be determined if below AI [20,21].

### 2.6. Comparison of Food-Based VLED to Formula VLED

To determine the nutritional equivalence of the diets to formula VLEDs in general, diets were compared to the “*Codex Alimentarius* standard for formula foods for use in very low energy diets for weight reduction” (CXS 203-1995) [5]. Food-based diets were also compared to the analyzed content of Optifast^®^ VLCD (Nestlé Health Science, Rhodes, NSW, Australia), as it serves as a specific example of formula VLED that is widely recognized and used in clinical practice. It is also a well-established product that has been extensively studied. Optifast^®^ was analyzed using publicly available nutrient composition values published online [22]. The average content of all product types (e.g., shakes, bars, desserts and soups) was combined proportionally based on the consumption of 3 products per day using the same method as Gibson et al., 2016 [9], which did not include additional allowed foods such as non-starchy vegetables, oils or beverages.

### 2.7. Data Analysis

For nutrients with EAR, data were reported as the difference to EAR and in terms of probability of adequacy or inadequacy as determined according to the Institute of Medicine’s Dietary Reference Intakes: Applications in Dietary Assessment [21]. To determine the probability, the difference (D) between the diet and EAR (D=diet−EAR) was divided by the standard deviation of D (SD_D_) to account for intra- and inter-person variations, resulting in the ratio $D/{SD}_{D}$. As per the Institute of Medicine, this ratio corresponded to a specific probability of adequacy or inadequacy, and was interpreted as having “high”, “moderate” or “low” for probability values ≥85%, 70% and 50%, respectively (Table 1). A positive $D/{SD}_{D}$ ratio indicated adequacy, while a negative $D/{SD}_{D}$ ratio indicated inadequacy. For nutrients with AI, data were reported as the difference to AI (diet−AI) and percentage of the AI (diet/AI×100). Comparisons with formula VLEDs were presented as proportions of the reference value.

## 3. Results

### 3.1. Diets Identified for Analysis

The scientific literature search identified a total of 775 titles, including 4 additional titles found from manual searching. After removing 180 duplicates, titles and abstracts were screened and another 433 articles were excluded. Full texts for the remaining articles were assessed for eligibility for inclusion. The corresponding authors of 40 articles were contacted to supply a detailed menu, of which only 1 responded. Thus, a total of 161 articles did not meet inclusion criteria, with reasons indicated in Figure 1, resulting in only one diet suitable for inclusion. An additional eight diets were identified from the online search. In total, nine food-based VLEDs were analyzed [23,24,25,26,27,28,29,30,31]. Seven diets were based on a low-carbohydrate Mediterranean way of eating, of which two were specifically designed to have carbohydrate <50 g/day [30,31]. One diet was a hospital-prescribed diet used in the pre-operative bariatric surgery setting [25]. The remaining diet was a compilation of low-calorie recipes from a website without any particular focus on nutrition or specific dietary pattern [28]. A brief description of each diet is presented in Table 2.

### 3.2. Nutrient Composition of the Diets

The mean nutrient composition of the nine food-based VLEDs and Optifast^®^ are shown in Table 3. Energy content ranged between 676 and 1157 kcal/day (2.8–4.8 MJ/day), despite all diets being described as having ≤800 kcal/day. Only two diets strictly met the criteria for VLED (≤800 kcal/day) [27,28]. Four diets ranged between 800 and 900 kcal/day [23,24,26,29], while the remaining three diets exceeded 1000 kcal/day [25,30,31]. Optifast^®^ provided the least energy at 619 kcal/day (2.6 MJ/day). Seven of ten food-based VLEDs contained carbohydrate <50 g/day [24,26,27,28,29].

### 3.3. Nutritional Adequacy of the Diets (Comparison with Nutrient Reference Values)

#### 3.3.1. Nutrients with EAR Values

The differences between VLEDs in nutrients with EARs are presented in Table 4. Calculated probabilities of adequacy and inadequacy are presented in Supplementary Materials (Table S4). Among the food-based diets, none provided all nutrients at or above the EAR. One diet [25] was slightly below EAR for iron (−1 mg) for women, and thiamine (−0.1 mg), magnesium (−57 mg) and zinc (−4.3 mg) for men, all corresponding to a low (50%) probability of inadequacy. 

Among the diets furthest from meeting EARs, one diet [28] had at least three nutrients at a high (≥85%) probability of inadequacy for both sexes. This diet was sourced from a recipe book that compiled recipes from a popular recipe website, and categorized them as 100 kcal, 250 kcal or 500 kcal, so readers can create their own menu plan combinations. It had no particular dietary pattern or nutritional focus (Table 2). Another two diets [27,29] had at least three nutrients at a high probability of inadequacy, but for men only. Both were companion recipe books derived from diet principles in another source [26]. These three diets belong in the same series of publications (i.e., “*The Fast 800*” diet book [26] and two companion recipe books [27,29]), and are self-described as being a low-carbohydrate Mediterranean style diet [26,27,29]. They encourage minimizing or avoiding white starchy grains/cereals (e.g., bread, pasta, potatoes, rice), tropical fruits and processed foods (Table 2).

The remaining food-based diets [23,24,30,31] had up to three nutrients for women and four nutrients for men at a moderate (70–85%) or high probability of inadequacy. Two of these diets [30,31] were self-described as ketogenic diets and aimed for <50 g/day of carbohydrate (Table 2). While the analyzed content of both these self-proclaimed ketogenic diets [30,31] was indeed <50 g/day for carbohydrate, most other diets [24,26,27,28,29] also had an analyzed carbohydrate content of <50 g/day (Table 3) but did not describe themselves as being ketogenic diets.

The most common nutrients at a high (≥85%) confidence of inadequacy included magnesium, calcium and iodine. Zinc was also inadequate for men in most food-based diets at a moderate (70%) confidence [24,26,27,28,29,30,31]. Optifast^®^ provided all nutrients above the EAR, with most nutrients at a high probability (≥85%) of adequacy. 

#### 3.3.2. Nutrients with AI Values

The differences between VLEDs in nutrients with AIs are presented in Table 5. Nutrients as a percentage of AI are presented in Supplementary Materials (Table S5). Among food-based diets, one diet [23] provided all nutrients at or above AI except two nutrients (potassium and linoleic acid) in men. This diet also exceeded the AI for dietary fiber. All other food-based diets provided between 50 and 99% of AI for dietary fiber. There was one [27] that fell short by 18 g for men and 13 g for women. Similarly, Optifast^®^ provided 17 g and 12 g less than the AI for dietary fiber in men and women, respectively (Table 5).

Among diets furthest from meeting the AI for all nutrients, two diets [25,28] supplied below 50% of AI for the essential fatty acids. One of these diets [25] also supplied all other nutrients below the AI, except potassium in women.

All VLEDs compared (food-based and Optifast^®^), met or exceeded AI for vitamin E and sodium, except one [25]. All diets also provided >50% of AI for potassium. Essential fatty acid (linoleic acid and α linolenic acid) content varied among food-based VLEDs. Some food-based diets provided >50% of AI for both sexes [23,24,30,31], while others were <50% of AI for one or both sexes. Optifast^®^ provided <50% of AI for linoleic acid for both sexes, and α linolenic acid (in men only). 

### 3.4. Comparison with Standards for Formula VLEDs

#### 3.4.1. Comparison with *Codex Alimentarius* Standard (CXS 203-1995)

None of the food-based diets nor Optifast^®^ satisfied all nutrient criteria described by the *Codex Alimentarius* standard (CXS 203-1995) (Table 6). Among the food-based VLEDs, one diet [23] had the fewest (three nutrients) below 100% of criteria, but one of these nutrients (iodine) was supplied at 43% of the criterion. The remaining diets [24,26,30] had up to eight nutrients that did not meet criteria, but none were at levels below 50% of criteria. 

Among the diets furthest from achieving all nutrient criteria, 1 diet [27] had 11 nutrients below criteria, of which 3 nutrients (carbohydrate, iron, iodine) were <50% of the criteria. Similarly, another diet (a recipe booked derived from the principles of a self-described ketogenic diet) [31], had three nutrients (carbohydrate, vitamin B6, iodine) below 50% of the criteria; however, only a total of eight nutrients were below 100% of the criteria. The remaining diets [25,28,29] had 7 to 10 nutrients below criteria.

**Table 4:** Difference between Optifast^®^ and 9 food-based VLEDs and EAR for adult males and females 19–50 years (diet minus EAR).

Nutrient	Optifast^®^		Mosley 2015 [23]		Bailey 2016 [24]		Baldry 2017 [25]		Mosley 2019 [26]		Bailey 2019 [27]		Myers-Cooke 2020 [28]		Bailey 2021 [29]		Mosley 2021 [30]		Bailey 2022 [31]	
	M	F	M	F	M	F	M	F	M	F	M	F	M	F	M	F	M	F	M	F
Protein (g)	8	23	10	25	17	32	25	40	3	18	9	24	0	15	11	26	12	27	17	32
Thiamine (mg)	0.6	0.7	−0.2	−0.1	−0.2	−0.1	−0.1	0	−0.2	−0.1	−0.2	−0.1	−0.2	−0.1	−0.4	−0.3	−0.2	−0.1	−0.2	−0.1
Riboflavin (mg)	1.2	1.4	−0.2	0	−0.2	0	1.2	1.4	−0.1	0.1	−0.4	−0.2	−0.3	−0.1	−0.3	−0.1	0	0.2	−0.1	0.1
Niacin equiv. (mg)	16	17	19	20	20	21	19	20	11	12	18	19	10	11	21	22	13	14	15	16
Vitamin C (mg)	91	91	282	282	294	294	137	137	149	149	116	116	137	137	184	184	145	145	74	74
Vitamin B6 (mg)	1.8	1.8	1.3	1.3	0.7	0.7	0.1	0.1	0.6	0.6	0.3	0.3	−0.1	−0.1	0.3	0.3	0.5	0.5	−0.1	−0.1
Vitamin B12 (µg)	1.7	1.7	0.5	0.5	1	1	2.8	2.8	1.4	1.4	0.1	0.1	−0.2	−0.2	0.6	0.6	1.2	1.2	0.8	0.8
Folate equiv. (µg)	274	274	237	237	235	235	187	187	113	113	−6	−6	81	81	−24	−24	0	0	10	10
Vitamin A equiv. (µg)	414	539	1195	1320	781	906	320	445	177	302	443	568	697	822	238	363	95	220	156	281
Magnesium (mg)	116	201	−22	63	−78	7	−57	28	−126	−41	−146	−61	−150	−65	−159	−74	−119	−34	−144	−59
Calcium (mg)	541	541	−18	−18	−250	−250	246	246	−449	−449	−562	−562	−518	−518	−546	−546	−288	−288	−443	−443
Phosphorus (mg)	647	647	595	595	478	478	777	777	378	378	275	275	190	190	331	331	443	443	433	433
Iron (mg)	17	15	13	11	4	2	1	−1	4	2	1	−1	3	1	1	−1	2	0	2	0
Zinc (mg)	1.7	7.2	−3.5	2	−5.5	0	−4.3	1.2	−5.7	−0.2	−6.9	−1.4	−5.8	−0.3	−5.8	−0.3	−6	−0.5	−4.8	0.7
Selenium (µg)	52	62	12	22	16	26	1	11	6	16	4	14	−23	−13	6	16	5	15	0	10
Iodine (µg)	169	169	−40	−40	−9	−9	63	63	2	2	−51	−51	−47	−47	−49	−49	−17	−17	−46	−46

Note: M indicates male. F indicates female. Red shading indicates high confidence (≥85%) of nutritional inadequacy, assuming long-term usual intake of the diet. Yellow shading indicates moderate confidence (70–85%) of nutritional inadequacy, assuming long-term usual intake of the diet. Unshaded indicates low confidence (50%) of nutritional adequacy or inadequacy.

**Table 5:** Difference between Optifast^®^ and 9 food-based VLEDs and AI for adult males and females 19–50 years (diet minus AI).

Nutrient	Optifast^®^		Mosley 2015 [23]		Bailey 2016 [24]		Baldry 2017 [25]		Mosley 2019 [26]		Bailey 2019 [27]		Myers-Cooke 2020 [28]		Bailey 2021 [29]		Mosley 2021 [30]		Bailey 2022 [31]	
	M	F	M	F	M	F	M	F	M	F	M	F	M	F	M	F	M	F	M	F
Dietary fiber (g)	−17	−12	1	6	−7	−2	−9	−4	−12	−7	−18	−13	−10	−5	−13	−8	−10	−5	−15	−10
Vitamin E (mg)	11	14	11	14	9	12	−4	−1	4	7	3	6	0	3	4	7	3	6	3	6
Sodium (mg)	449–909	449–909	679–1139	679–1139	1201–1661	1201–1661	−460	−460	919–1379	919–1379	122–582	122–582	39–499	39–499	591–1051	591–1051	696–1156	696–1156	998–1458	998–1458
Potassium (mg)	−1084	−84	−603	397	−887	113	−681	319	−1273	−273	−1735	−735	−1696	−696	−1605	−605	−1512	−512	−1556	−556
Linoleic acid	−11	−6	−3	2	−6	−1	−10	−5	−7	−2	−8	−3	−9	−4	−8	−3	−5	0	−5	0
α linolenic acid	−0.7	−0.2	1.6	2.1	−0.5	0	−1	−0.5	−0.2	0.3	−0.4	0.1	−1	−0.5	−0.6	−0.1	−0.1	0.4	−0.2	0.3

Note: M indicates male. F indicates female. Red shading indicates amounts <50% of AI. Yellow shading indicates amounts between 50% and 99% of AI. Unshaded cells indicate amounts ≥100% of AI.

**Table 6:** Mean energy and nutrient provision of Optifast^®^ and 9 food-based VLED as a proportion (%) of the *Codex Alimentarius* standard (CXS 203-1995) [5].

Nutrient	Criteria, Units Per Day	Optifast^®^	Mosley 2015 [23]	Bailey 2016 [24]	Baldry 2017 [25]	Mosley 2019 [26]	Bailey 2019 [27]	Myers-Cooke 2020 [28]	Bailey 2021 [29]	Mosley 2021 [30]	Bailey 2022 [31]
Energy	450–800 kcal	77	122	124	133	121	96	84	104	131	145
Protein	>50 g	120	125	137	154	111	122	104	127	128	137
Carbohydrate	>50 g	115	101	72	237	66	49	95	63	82	45
Thiamine	>0.8 mg	206	100	100	107	94	95	103	81	94	101
Riboflavin	>1.2 mg	188	72	76	192	80	62	67	69	92	84
Niacin equiv.	>11 mg	251	284	289	279	209	273	200	296	223	248
Vitamin E	>10 mg	208	206	186	62	141	130	101	143	126	127
Vitamin B6	>2 mg	147	122	89	58	83	71	51	72	80	48
Vitamin B12	>1 µg	374	252	300	482	339	205	177	255	321	282
Folate equiv.	>200 µg	178	167	167	152	130	94	120	89	96	99
Vitamin A equiv.	>600 µg	173	303	234	157	134	178	220	144	120	130
Sodium	>1000 mg	137	160	212	81	184	104	96	151	162	192
Potassium	>1600 mg	170	200	182	195	158	129	131	137	143	140
Magnesium	>350 mg	133	94	78	84	64	58	57	55	66	59
Calcium	>500 mg	276	164	118	217	78	56	64	59	110	79
Phosphorus	>500 mg	245	235	212	271	192	171	154	182	205	203
Iron	>16 mg	147	116	61	45	64	43	55	44	52	51
Zinc	>6 mg	229	141	109	128	105	86	104	103	100	120
Iodine	>140 µg	192	43	65	117	73	35	38	37	59	39
Linoleic acid	>3 g	76	350	223	84	208	174	118	176	267	283
α linolenic acid	<0.5 g	123	585	164	70	214	173	64	139	240	218

Note: Red shading indicates amounts <50% of the minimum *Codex Alimentarius* nutrient criteria (or higher than the maximum criteria, applicable to α linoleic acid only). Yellow shading indicates amounts between 50% and 99% of the minimum *Codex Alimentarius* nutrient criteria. Unshaded cells indicate amounts ≥100% of the minimum *Codex Alimentarius* nutrient criteria (or lower than the maximum criteria, applicable to α linoleic acid only).

The most common nutrients supplied at less than 100% of these criteria included magnesium (all diets), riboflavin, vitamin B6, iron and iodine (eight diets) and calcium (five diets). Iodine was supplied at <50% of the criteria for five diets [23,27,28,29,31]. Optifast^®^ was low only for linoleic acid (76% of criteria); however, it exceeded all other nutrient criteria.

#### 3.4.2. Comparison with Optifast^®^

None of the food-based VLEDs had the exact same nutrient profile as Optifast^®^ (Table 3). The 2 diets [23,25] closest to the nutrient profile of Optifast had 13 nutrients supplied at levels below Optifast^®^. In one of these diets [23], two nutrients (riboflavin and iodine) were supplied below 50% of Optifast^®^. The other diet [25] had three nutrients (vitamin E, vitamin B6, iron) supplied below 50% of Optifast^®^. All remaining diets [24,26,27,28,29,30,31] were not similar to the nutrient profile of Optifast^®^ and had at least four nutrients supplied below 50% of Optifast^®^.

Six out of nine food-based VLEDs had greater quantities of energy, dietary fiber, protein, total fat and linoleic acid compared with Optifast^®^. Conversely, Optifast^®^ was higher in carbohydrate than all food-based VLEDs, except one [25] (Table 3). At least 1 food-based VLED had greater or equal amounts of the following 9 (out of 18) micronutrients compared with Optifast^®^: riboflavin [21], niacin [21,23,24,25,28], vitamin C [21,23,24,25,26,27,28,29], vitamin E [23], vitamin B12 [21], vitamin A [23,24,25,27], sodium [23,24,26,28,29,30], potassium [21,23,24] and phosphorus (21). Optifast^®^ was higher than all food-based VLEDs for the remaining nine micronutrients, including thiamine, vitamin B6, folate, magnesium, calcium, iron, zinc, selenium and iodine (Table 3). Most food-based VLEDs provided <50% of Optifast^®^ for minerals and between 50 and 99% of Optifast^®^ for vitamins (Supplementary Materials, Table S6).

## 4. Discussion

The potential for food-based VLEDs to be clinically recommended as an alternative to formula approaches is largely limited by the belief that food-only diets cannot be nutrient-dense enough to supply adequate nutrition within an 800 kcal/day limit. No analysis of food-based VLEDs has previously been performed to examine their nutritional adequacy. The main findings of this comparison of nine food-based VLEDs were that they were inadequate in several micronutrients, including magnesium, calcium, iodine and zinc for men, despite often exceeding 800 kcal per day. They did, however, offer greater quantities and varieties of dietary fiber than formula VLEDs, suggesting potential advantages in terms of dietary fiber intake.

To our knowledge, there has been no previous comparison of contemporary food-based VLEDs with formula VLEDs. Most studies of food-based approaches to VLED date back to the 1980s and 1990s, before formula VLEDs became the default approach [32,33,34,35,36,37,38,39,40,41,42,43,44,45,46,47,48,49,50,51,52,53]. The protein-sparing modified fast (PSMF), one of the more well-known types of food-based VLEDs of this era that originated in 1978 [54], is reported to have similar nutritional deficits to the diets analyzed here. This PSMF was not included in this analysis because it is unbalanced (i.e., consisting of foods from only two of five core food groups in the Australian Dietary Guidelines), and requires supplementation with multiple vitamins and minerals, including potassium, magnesium, calcium and sodium [55]. 

In addition to the known nutrient deficits in the PSMF, our results indicate that balanced and non-supplemented food-based VLEDs are likely to have iodine deficits, especially when full-fat varieties of dairy (milk, yoghurt, cheese) are used. Our analysis shows that VLEDs adequate in iodine comprised 33% of total calories from low-fat or skimmed dairy products, while diets allowing full-fat dairy consisted of approximately 10% of total calories from dairy foods. As low-fat and skimmed dairy products are more nutrient-dense and less calorie-dense than full-fat dairy products, greater amounts can be included to increase micronutrient levels, such as iodine, while minimizing calories. Thus, choosing low-fat and skimmed dairy products is an important strategy for meeting iodine requirements in food-based VLEDs.

Inadequate intakes of essential nutrients can have negative health implications, especially if inadequate intake is prolonged over many months to years. These include osteoporosis for calcium and magnesium [56,57], hypothyroidism and goiter for iodine [58] and frequent infections and hypogonadism in men for zinc [59,60]. However, VLEDs are typically taken for 8 to 16 weeks [1], a relatively brief period, during which clinically important deficiencies are unlikely to arise. Nonetheless, it is important that patients undertaking food-based VLEDs do so under the care of a dietitian, who can monitor and address any risks of nutritional inadequacies.

Furthermore, food-based VLEDs have the potential to be recommended in clinical settings, provided that the identified nutrient deficits (magnesium, calcium, iodine, zinc and potassium) are addressed. Various approaches can be taken to achieve this. One is to add intrinsically micronutrient-dense food sources such as meat, seafood and dairy instead of wholegrains and cereals, which contain relatively low amounts of these nutrients. Greater amounts of low-starch vegetables will also increase potassium and dietary fiber. But a food-based approach will inevitably affect the intake of energy and other nutrients. Even if it is possible to manipulate the diet to meet all nutrient benchmarks, the diet may become infeasible or expensive to implement, contain unusual and unpalatable combinations of food ingredients and be unacceptable to potential users.

A more convenient approach is to use vitamin and/or mineral supplements. Potassium and iodine may be easily added with minimal impact on energy by allowing ad lib use of potassium-iodide salt to season food. These are commonly known as “lite salt”, “low-sodium salt” or “salt substitutes” and are readily available from supermarkets. The remaining nutrients (magnesium, calcium and zinc) could be attained from taking multivitamin/multimineral supplements that contain adequate quantities of the deficient micronutrients.

Food-based VLEDs offer several potential advantages over formula VLEDs. They provide higher quantities and a broader variety of dietary fiber, which is associated with lower risks of obesity-related comorbidities including cardiovascular diseases (coronary heart disease, stroke), type 2 diabetes mellitus and certain cancers (colorectal and rectal) [10,11]. Dietary fiber exists in various forms naturally found in vegetables, fruit, legumes and whole grains, such as cellulose, hemicellulose, pectin, resistant starch, inulin and oligofructose and lignin [61]. These different types of fibers offer various functional benefits, including delayed gastric emptying, improved glycemic control, laxation and lowering cholesterol levels [61]. In contrast, formula VLEDs provide less than 50% of AI for dietary fiber and may contain fermentable oligosaccharides, disaccharides and monosaccharides and polyols (FODMAPs) [22]. FODMAPs can trigger gastrointestinal symptoms in individuals with irritable bowel syndrome [62]. Moreover, food-based VLEDs can be tailored to accommodate individual food preferences, making them advantageous for those who cannot tolerate or prefer not to use meal replacement products. Some studies have reported that food-based VLEDs are more acceptable and enjoyable than formula diets, with participants reporting lower levels of hunger and social disruptiveness compared with those on the formula VLED [42].

Formula VLEDs also offer distinct advantages. They are formulated to provide all essential nutrients in specific proportions, ensuring a nutritionally complete diet for all nutrients within the 800 kcal/day limit. Users find them convenient and easy-to-use as they require little to no preparation or decision making around meals [7]. Extensive research supports the efficacy and safety of formula VLEDs, with studies indicating that individuals on formula VLEDs are more likely to achieve weight-loss goals and are less likely to report difficulty in adhering to the diet compared with those on isocaloric food-based diets [6].

There are several limitations to this analysis. This was not an original study evaluating different diets in a population under consistent criteria. The nutrient data reflect the theoretical nutrient composition of each diet and represent intake at full adherence to the analyzed menus. Given the theoretical nature of this work, it did not consider real-world factors that influence intake, including patient adherence, personal preferences or access to the required food or ingredients. Adherence to dietary intervention is a crucial factor in determining their effectiveness and acceptability of the diet. Additionally, the potential effects of these diets on short- and long-term weight status, obesity-related comorbidities and overall health outcomes were not explored in this work.

Another limitation is that nutrient reference values serve as a valid benchmark for assessing the likelihood of nutritional adequacy or inadequacy only when nutrient intakes represent long-term usual consumption. Given this necessary assumption, short-term intakes that fall below the nutrient reference values do not necessarily indicate nutritional inadequacy. In this study, only 7 days’ worth of menus were analyzed, which may not be representative of long-term intakes. Ideally, benchmark nutrient values for assessing nutritional adequacy of VLEDs should be tailored for short-term diets. While the *Codex Alimentarius* standard for formula VLEDs serves this purpose for formula VLEDs, it expressly excludes application to food-based VLEDs. The *Codex Alimentarius* also does not specify values for dietary fiber nor some essential micronutrients, including vitamin C and selenium. Consequently, assessing the nutritional adequacy of short-term food-based diets remains challenging, highlighting the need for more comprehensive guidance.

Lastly, while we used a consistent procedure for analyzing the selected diets in this review, it is important to acknowledge that the original nutrition analyses reported for each diet likely used different methodologies to ours. For example, items such as beverages, condiments and “allowed extras” (such as low-starch vegetables) in the formula VLED were not included and therefore may underestimate dietary fiber and micronutrients offered by strict adherence to the Optifast^®^ protocol. The computerized nutritional analysis approach used also does not account for variations due to factors such as seasons, procurement, production or duration of cooking, which can influence the nutritional content of the foods analyzed, and thereby affect the resulting nutritional content of the diet.

## 5. Conclusions

In conclusion, despite the common perception that formulated meal replacements are necessary if a VLED is prescribed, a balanced food-based VLED could potentially be used in a clinical setting provided deficits in magnesium, calcium, iodine and zinc are overcome through supplementation or dietary manipulation. Both food and formula approaches have their unique advantages and limitations. Food-based VLEDs offer the advantage of higher quantities and greater varieties of dietary fiber, while formula VLEDs provide greater certainty around nutritional adequacy while also being convenient and easy to use and prepare. Thus, recommendations for either approach should consider individuals’ preferences, tolerance of the specific foods or products and adherence to the diet.

Future studies should directly compare short- and long-term outcomes of food and formula approaches to VLED. Well-designed clinical trials are warranted to evaluate adherence, weight-loss outcomes, changes in obesity-related comorbidities and overall health implications of both approaches. Such investigations would provide valuable information to guide the development of effective dietary strategies for the management of obesity.

## Figures and Tables

**Table 1:** Interpretation of estimated average requirement (EAR) according to the difference/standard deviation of the difference (D/SD_D_) ratio and probability of correctly concluding that usual intake is adequate or inadequate.

D/SD_D_ Criterion	Probability of Correct Conclusion	Interpretation ^1^
>2.00	98%	High (A)
>1.65	95%	High (A)
>1.50	93%	High (A)
>1.00	85%	High (A)
>0.05	70%	Moderate (A)
>0.00	50%	Low (A) or (I)
<−0.50	70%	Moderate (I)
<−1.00	85%	High (I)
<−1.50	93%	High (I)
<−1.65	95%	High (I)
<−2.00	98%	High (I)

^1^ Interpretation reads “there is a high/moderate/low likelihood that usual intake is adequate (A)/inadequate (I)”. Adapted from the Institute of Medicine (2000), Table B-1 [21].

**Table 2:** Author, title and description of diets included for menu analysis.

Author	Title	Description
Mosley 2015 [23]	The 8-Week Blood Sugar Diet (Australian and New Zealand Edition)	Self-described as a low-carbohydrate Mediterranean style diet. Minimize or avoid sugar, treats, desserts, sweet drinks; white starchy grains/cereals (e.g., bread, pasta, potatoes, rice); tropical fruits and margarine. Swap these for wholegrain varieties, low-carbohydrate fruits (e.g., berries, apples) and butter. Encourage full-fat dairy. Allow wine or spirits in moderation but not beer [23].
Bailey 2016 [24]	The 8-Week Blood Sugar Diet Recipe Book	Recipes and menus based on [23].
Baldry 2017 [25]	Derby Teaching Hospitals, NHS Foundation Trust Standard Pre-Op Diet for Bariatric Surgery	Three meals per day. No sugar or oils/fats allowed. All foods need to be weighed.
Mosley 2019 [26]	The Fast 800: How to Combine Rapid Weight Loss and Intermittent Fasting for Long-Term Health (Australian and New Zealand Edition)	Self-described as a low-carbohydrate Mediterranean style diet. Minimize or avoid white starchy grains/cereals (e.g., bread, pasta, potatoes, rice), tropical fruits and processed foods. Swap these for wholegrains and pulses, low-carbohydrate fruits (e.g., berries, apples). Encourage “natural healthy fats” (e.g., olive oil, salmon, tuna, full-fat dairy, avocado, nuts and seeds) and plenty of green and colored vegetables. Discourage snacks and grazing. No alcohol [26].
Bailey 2019 [27]	The Fast 800 Recipe Book (Australian and New Zealand Edition)	Recipes and suggested menus based on [26].
Myers-Cooke 2020 [28]	The Fast Revolution: the best of the best recipes from Australians’ #1 food site	A compilation of recipes categorized as 100 kcal, 250 kcal and 500 kcal. Main focus is caloric content; no particular dietary pattern or nutritional focus otherwise. Readers create their own menu plans from combinations of the recipe categories, e.g., Option A: 500 kcal + 250 kcal + 100 kcal; Option B: 250 kcal + 250 kcal + 250 kcal
Bailey 2021 [29]	The Fast 800 Easy (Australian and New Zealand Edition)	Self-described as a “lowish” carbohydrate Mediterranean style diet using easily accessible foods found in pantry or freezer, not necessarily fresh produce. Discourages starchy and processed foods, sugars, sweeteners. Encourages protein, non-starchy vegetables, low-carbohydrate fruits, plant-based fats (e.g., canola, olive oil), full-fat dairy and grass-fed meats.
Mosley 2021 [30]	The Fast 800 Keto: Eat Well, Burn Fat, Manage Your Weight Long-Term	Similar to [23,26], except aims for protein >50 g/day and carbohydrate <50 g/day. Protein at every meal, in moderation, 2 or 3 meals per day.
Bailey 2022 [31]	The Fast 800 Keto Recipe Book: Delicious Low-Carb Recipes for Rapid Weight Loss And Long-Term Health	Recipes and suggested menus based on [30].

**Table 3:** Mean energy and nutrient provision of Optifast^®^ and 9 food-based VLEDs.

Nutrient	Optifast^®^	Mosley 2015 [23]	Bailey 2016 [24]	Baldry 2017 [25]	Mosley 2019 [26]	Bailey 2019 [27]	Myers-Cooke 2020 [28]	Bailey 2021 [29]	Mosley 2021 [30]	Bailey 2022 [31]
Energy (MJ)	2.6	4.1	4.2	4.4	4.1	3.2	2.8	3.5	4.4	4.8 *
Energy (kcal)	619	975	991	1062	969	771	676	829	1044	1157 *
Protein (g)	60	62	69	77 *	55	61	52	63	64	69
Total fat (g)	15	49	59	24	64	45	26	46	65	85 *
- saturated (g)	4	12	16	8	20	11	7	10	20	24 *
- poly (g)	1	14 *	8	4	8	7	4	7	10	10
- mono (g)	2	19	29	9	31	23	12	25	29	45 *
Carbohydrate (g)	57	51	36	118 *	33	25	47	32	41	23
Dietary fiber (g)	13	31 *	23	21	18	12	20	17	20	15
Thiamine (mg)	1.6 *	0.8	0.8	0.9	0.8	0.8	0.8	0.6	0.8	0.8
Riboflavin (mg)	2.3 *	0.9	0.9	2.3 *	1	0.7	0.8	0.8	1.1	1
Niacin equiv. (mg)	28	31	32	31	23	30	22	33 *	25	27
Vitamin C (mg)	121	312	324 *	167	179	146	167	214	175	104
Vitamin E (mg)	21 *	21 *	19	6	14	13	10	14	13	13
Vitamin B6 (mg)	2.9 *	2.4	1.8	1.2	1.7	1.4	1	1.4	1.6	1
Vitamin B12 (µg)	3.7	2.5	3	4.8 *	3.4	2.1	1.8	2.6	3.2	2.8
Folate equiv. (µg)	594 *	557	555	507	433	314	401	296	320	330
Vitamin A equiv. (µg)	1039	1820 *	1406	945	802	1068	1322	863	720	781
Sodium (mg)	1369	1599	2121 *	813	1839	1042	959	1511	1616	1918
Potassium (mg)	2716	3197 *	2913	3119	2527	2065	2104	2195	2288	2244
Magnesium (mg)	466 *	328	272	293	224	204	200	191	231	206
Calcium (mg)	1381 *	822	590	1086	391	278	322	294	552	397
Phosphorus (mg)	1227	1175	1058	1357 *	958	855	770	911	1023	1013
Iron (mg)	23 *	19	10	7	10	7	9	7	8	8
Zinc (mg)	13.7 *	8.5	6.5	7.7	6.3	5.1	6.2	6.2	6	7.2
Selenium (µg)	112 *	72	76	61	66	64	37	66	65	60
Iodine (µg)	269 *	60	91	163	102	49	53	51	83	54
Linoleic acid(g)	2	10 *	7	3	6	5	4	5	8	8
α linolenic acid (g)	0.6	2.9 *	0.8	0.3	1.1	0.9	0.3	0.7	1.2	1.1

* Indicates highest content across all diets in this table (food-based and Optifast^®^). Red shading indicates amounts <50% of Optifast^®^. Yellow shading indicates amounts between 50% and 99% of Optifast^®^. Unshaded cells indicate amounts ≥100% of Optifast^®^.

## Data Availability

The original contributions presented in this study are included in the article/Supplementary Materials, further inquiries can be directed to the corresponding authors.

## Supplementary Materials

The following supporting information can be downloaded at: https://www.mdpi.com/article/10.3390/nu16172993/s1, Table S1: Comparison of *Codex Alimentarius* standard (CXS 203-1995) and Australian nutrient reference values (EAR, RDI/AI) for adult males and females 19–50 years; Table S2: Complete search strategy and search terms; Table S3: Standard amounts entered for nonspecific measures of foods; Table S4: Probability (%) of adequacy or inadequacy (i) of Optifast^®^ and 9 food-based VLEDs for adult males (M) and females (F) 19–50 years; Table S5: Nutrient content of Optifast^®^ and 9 food-based VLEDs as a proportion (%) of AI for adult males and females 19–50 years (Diet/AI x100); Table S6: Mean energy and nutrient provision of 9 food-based VLED as a proportion (%) of Optifast^®^.
