# Supplementary material for: Comparison of the Nutritional Adequacy of Current Food-Based Very Low Energy Diets: A Review and Nutritional Analysis
Source: Nutrients. 2024 Sep 5;16(17):2993. doi: 10.3390/nu16172993 (PMC11396843; doi:10.3390/nu16172993)
Supplement: Supplementary file 1 [file nutrients-16-02993-s001.zip › Poon_Nutrients Supp S1.pdf]

Table S1. Comparison of Codex Alimentarius standard (CXS 203-1995) and Australian Nutrient Reference Values (EAR, RDI/AI) for adult males and females 19-50 years.

Energy and Nutrients	Codex Alimentarius standard	Men 19-50 years		Women 19-50 years	
		EAR	RDI/AI*	EAR	RDI/AI*
Energy (MJ)	1.9-3.3	-	-	-	-
Energy (kcal)	450-800	-	-	-	-
Protein (g)	>50	52	64	37	46
Total fat (g)	-	-	-	-	-
- Saturated (g)	-	-	-	-	-
- Polyunsaturated (g)	-	-	-	-	-
- Monounsaturated (g)	-	-	-	-	-
- Linoleic acid (g)	>3	N/A	13*	N/A	8*
- alpha linolenic acid (g)	<0.5	N/A	1.3*	N/A	0.8*
Carbohydrate (g)	>50	-	-	-	-
Dietary fibre (g)	-	N/A	30*	N/A	25*
Thiamine (mg)	>0.8	1.0	1.2	0.9	1.1
Riboflavin (mg)	>1.2	1.1	1.3	0.9	1.1
Niacin equivalents (mg)	>11	12	16	11	14
Vitamin C (mg)	-	30	45	30	45
Vitamin E (mg)	>10	N/A	10*	N/A	7*
Vitamin B6 (mg)	>2	1.1	1.3	1.1	1.3
Vitamin B12 (μg)	>1	2.0	2.4	2.0	2.4
Folate equivalents (μg)	>200	320	400	320	400
Vitamin A equivalents (μg)	>600	625	900	500	700
Vitamin D (ug)	>2.5	N/A	5*	N/A	5*
Sodium (mg)	>1000	N/A	420-920*	N/A	420-920*
Potassium (mg)	>1600	N/A	3800*	N/A	2800*
Magnesium (mg)	>350	220[a]-350[b]	400[a]-420[b]	255[a]-265[b]	310[a]-320[b]
Calcium (mg)	>500	840	1000	840	1000
Phosphorus (mg)	>500	580	1000	580	1000
Iron (mg)	>16	6	8	8	18
Zinc (mg)	>6	12	14	6.5	8
Selenium (μg)	-	60	70	50	60
Iodine (μg)	>140	100	150	100	150
Copper (mg)	>1.5	N/A	1.7*	N/A	1.2*

- Not set. N/A Not applicable. * Value is Adequate Intake (AI) as RDI has not been set for this nutrient. [a] 19-30 years only. [b] 31-50 years only.
