# Supplementary material for: Comparison of the Nutritional Adequacy of Current Food-Based Very Low Energy Diets: A Review and Nutritional Analysis
Source: Nutrients. 2024 Sep 5;16(17):2993. doi: 10.3390/nu16172993 (PMC11396843; doi:10.3390/nu16172993)
Supplement: Supplementary file 1 [file nutrients-16-02993-s001.zip › Poon_Nutrients Supp S2.pdf]

Tables S2. Complete search strategy and search terms.

Table S2-1. Full list of search terms used to identify published food-based VLEDs in the scientific literature.

Key term set no.	Search Terms	Number of articles retrieved
1	VLED* OR (very-low energy diet) OR (VLCD*) OR (very-low calorie diet) OR (800 kcal) OR (800 calorie) AND ((conventional food) or (normal food) or (natural food) or (food-based))	394
2	(PSMF) OR (Protein sparing modified fast)	269
3	(Semi starvation diet) OR (Semi-starvation diet) OR (Semistarvation diet)	108
	Total	771

Table S2-2 Summary of search outputs and article selection.

Database	Key terms set 1	Key terms set 2	Key terms set 3
PubMed	345	130	91
Ovid Medline	18	103	6
Cochrane Library	24	20	10
CINAHL Complete (EBSCO)	7	16	1
Hand searched	4	0	0
**Total retrieved**	**398**	**269**	**108**
	**TOTAL 775**		
Duplicates removed	35	131	14
Title/Abstract screened	363	138	94
Records excluded	287	89	57
Full text read	76	49	37
**Full text excluded, reasons below**	**75**	**49**	**37**
Starvation diet ≤ 200kcal	0	1	2
>800kcal	16	0	2
Used formula/meal replacement product, or other intervention	24	5	15
Unknown if food or formula diet	5	3	4
Not in English	3	4	0
Not Clinical trial (Review, commentary, case report, protocol)	9	22	7
No menu plan	18	14	7
**TOTAL included for menu analysis**	**1**	**0**	**0**

Table S2-3. Search queries for each database.

**Pubmed Search Queries**	
Key terms set 1	((VLED*) OR (very-low energy diet) OR (VLCD*) OR (very-low calorie diet) OR (800 kcal) or (800 calorie)) AND ((conventional food) or (normal food) or (natural food) or (food-based))
Key terms set 2	(PSMF) OR (protein sparing modified fast)
Key terms set 3	(semi starvation diet) OR (semi-starvation diet) OR (semistarvation diet))
**Ovid Medline Search Queries**	
Key terms set 1	
1	VLED.mp. [mp=title, book title, abstract, original title, name of substance word, subject heading word, floating sub-heading word, keyword heading word, organism supplementary concept word, protocol supplementary concept word, rare disease supplementary concept word, unique identifier, synonyms, population supplementary concept word, anatomy supplementary concept word] – 154 results from 23 Mar 2023
2	very-low energy diet.mp. [mp=title, book title, abstract, original title, name of substance word, subject heading word, floating sub-heading word, keyword heading word, organism supplementary concept word, protocol supplementary concept word, rare disease supplementary concept word, unique identifier, synonyms, population supplementary concept word, anatomy supplementary concept word] – 217 results from 23 Mar 2023
3	VLCD*.mp. [mp=title, book title, abstract, original title, name of substance word, subject heading word, floating sub-heading word, keyword heading word, organism supplementary concept word, protocol supplementary concept word, rare disease supplementary concept word, unique identifier, synonyms, population supplementary concept word, anatomy supplementary concept word] – 549 results from 23 Mar 2023
4	very-low calorie diet.mp. [mp=title, book title, abstract, original title, name of substance word, subject heading word, floating sub-heading word, keyword heading word, organism supplementary concept word, protocol supplementary concept word, rare disease supplementary concept word, unique identifier, synonyms, population supplementary concept word, anatomy supplementary concept word] – 728 results from 23 Mar 2023
5	800 kcal.mp. [mp=title, book title, abstract, original title, name of substance word, subject heading word, floating sub-heading word, keyword heading word, organism supplementary concept word, protocol supplementary concept word, rare disease supplementary concept word, unique identifier, synonyms, population supplementary concept word, anatomy supplementary concept word] – 417 results from 23 Mar 2023
6	800 calorie.mp. [mp=title, book title, abstract, original title, name of substance word, subject heading word, floating sub-heading word, keyword heading word, organism supplementary concept word, protocol supplementary concept word, rare disease supplementary concept word, unique identifier, synonyms, population supplementary concept word, anatomy supplementary concept word] - 20 results from 23 Mar 2023
7	conventional food.mp. [mp=title, book title, abstract, original title, name of substance word, subject heading word, floating sub-heading word, keyword heading word, organism supplementary concept word, protocol supplementary concept word, rare disease supplementary concept word, unique identifier, synonyms, population supplementary concept word, anatomy supplementary concept word] – 248 results from 23 Mar 2023
8	normal food.mp. [mp=title, book title, abstract, original title, name of substance word, subject heading word, floating sub-heading word, keyword heading word, organism supplementary concept word, protocol supplementary concept word, rare disease supplementary concept word, unique identifier, synonyms, population supplementary concept word, anatomy supplementary concept word] – 730 results from 23 Mar 2023
9	natural food.mp. [mp=title, book title, abstract, original title, name of substance word, subject heading word, floating sub-heading word, keyword heading word, organism supplementary concept word, protocol supplementary concept word, rare disease supplementary concept word, unique identifier, synonyms, population supplementary concept word, anatomy supplementary concept word] – 1848 results from 23 Mar 2023
10	food-based.mp. [mp=title, book title, abstract, original title, name of substance word, subject heading word, floating sub-heading word, keyword heading word, organism supplementary concept word, protocol supplementary concept word, rare disease supplementary concept word, unique identifier, synonyms, population supplementary concept word, anatomy supplementary concept word] – 1951 results from 23 Mar 2023

11	1 or 2 or 3 or 4 or 5 or 6 – 1528 results from 23 Mar 2023
12	7 or 8 or 9 or 10 – 4753 results from 23 Mar 2023
13	11 and 12 – 18 results from 23 Mar 2023
Key Terms set 2	
1	PSMF.mp. [mp=title, book title, abstract, original title, name of substance word, subject heading word, floating sub-heading word, keyword heading word, organism supplementary concept word, protocol supplementary concept word, rare disease supplementary concept word, unique identifier, synonyms, population supplementary concept word, anatomy supplementary concept word] – 55 results from 23 Mar 2023
2	protein-sparing modified fast.mp. [mp=title, book title, abstract, original title, name of substance word, subject heading word, floating sub-heading word, keyword heading word, organism supplementary concept word, protocol supplementary concept word, rare disease supplementary concept word, unique identifier, synonyms, population supplementary concept word, anatomy supplementary concept word] – 83 results from 23 Mar 2023
3	1 or 2 – 103 results from 23 Mar 2023
Key terms set 3	
1	Semi starvation diet.mp. [mp=title, book title, abstract, original title, name of substance word, subject heading word, floating sub-heading word, keyword heading word, organism supplementary concept word, protocol supplementary concept word, rare disease supplementary concept word, unique identifier, synonyms, population supplementary concept word, anatomy supplementary concept word] – 3 results from 23 Mar 2023
2	Semi-starvation diet.mp. [mp=title, book title, abstract, original title, name of substance word, subject heading word, floating sub-heading word, keyword heading word, organism supplementary concept word, protocol supplementary concept word, rare disease supplementary concept word, unique identifier, synonyms, population supplementary concept word, anatomy supplementary concept word] - 3 results from 23 Mar 2023
3	Semistarvation diet.mp. [mp=title, book title, abstract, original title, name of substance word, subject heading word, floating sub-heading word, keyword heading word, organism supplementary concept word, protocol supplementary concept word, rare disease supplementary concept word, unique identifier, synonyms, population supplementary concept word, anatomy supplementary concept word] – 3 results from 23 Mar 2023
4	1 or 2 or 3 – 6 results from 23 Mar 2023
**Cochrane Library Search Queries**	
Key terms set 1	(VLED* or "very-low-energy diet" or "very-low energy diet" or "very low-energy diet" or "very low energy diet" or VLCD* or "very-low-calorie diet" or "very-low calorie diet" or "very low-calorie diet" or “800 kcal” or 800 calorie*):ti,ab,kw AND ("Conventional food" or "normal food" or "natural food" or "food-based" or "food based"):ti,ab,kw (Word variations have been searched)
Key terms set 2	(PSMF):ti,ab,kw OR (psm):ti,ab,kw (Word variations have been searched)
Key terms set 3	(Semi starvation diet):ti,ab,kw OR (Semi-starvation diet):ti,ab,kw OR (Semistarvation diet):ti,ab,kw (Word variations have been searched)
**CINAHL Complete/ EBSCO Search Queries**	
Key terms set 1	
S1	VLED* OR very-low energy diet OR VLCD* OR very-low calorie diet OR 800 kcal OR 800 calorie – 505 results
S2	conventional food OR normal food OR natural food OR food-based – 2873 results
S3	(S1 AND S2) – 7 results
Key terms set 2	
S1	PSMF OR protein sparing modified fast – 16 results
Key terms set 3	
S1	Semi starvation diet OR Semi-starvation diet OR Semistarvation diet – 1 result
