# Supplementary material for: Comparison of the Nutritional Adequacy of Current Food-Based Very Low Energy Diets: A Review and Nutritional Analysis
Source: Nutrients. 2024 Sep 5;16(17):2993. doi: 10.3390/nu16172993 (PMC11396843; doi:10.3390/nu16172993)
Supplement: Supplementary file 1 [file nutrients-16-02993-s001.zip › Poon_Nutrients Supp S3.pdf]

Table S3. Standard amounts entered for nonspecific measures of foods.

Description	Standard weight entered
'Drizzle' of oil	7g
Oil 'for brushing'	2g
'Knob' of butter or margarine	7g
'Small knob' of butter or margarine	5g
'Large knob' of butter or margarine	10g
'Dash' of oil, tabasco or similar	7g
'Pinch' of spices or similar	0.4g
'Large pinch' of spices	0.8g
'A few sprigs' of fresh herbs or similar	3g
'Handful' of fresh herbs or similar	40g
'Small handful' of fresh herbs or similar	30g
'Large handful' of fresh herbs or similar	60g
'Handful' of raw vegetables leaves eg baby spinach or similar	75g
'Small handful' of raw vegetables leaves eg baby spinach or similar	30g
'Large' or 'generous handful' of raw vegetables leaves eg baby spinach or similar	100g
'Sprinkling' of seeds	20g
'Handful' of nuts	30g
'Handful' of grated cheese	40g
'Handful' of berries	150g
'Small handful' of berries	100g
'Juice' of a lemon or lime	40ml
'Splash' of lemon juice, sauce	10 ml
'Squeeze' of lemon or lime juice	10 ml
'Dollop' of chutney, cream	30g
