# Supplementary material for: Comparison of the Nutritional Adequacy of Current Food-Based Very Low Energy Diets: A Review and Nutritional Analysis
Source: Nutrients. 2024 Sep 5;16(17):2993. doi: 10.3390/nu16172993 (PMC11396843; doi:10.3390/nu16172993)
Supplement: Supplementary file 1 [file nutrients-16-02993-s001.zip › Poon_Nutrients Supp S4.pdf]

Table S4. Probability (%) of adequacy or inadequacy (i) of Optifast® and 9 food-based VLEDs for adult males (M) and females (F) 19-50 years.

Nutrient	Optifast®		Mosley 2015 [22]		Bailey 2016 [23]		Baldry 2017 [24]		Mosley 2019 [25]		Bailey 2019 [26]		Myers-Cooke 2020 [27]		Bailey 2021 [28]		Mosley 2021 [29]		Bailey 2022 [30]	
	M	F	M	F	M	F	M	F	M	F	M	F	M	F	M	F	M	F	M	F
Protein	50%	70%	50%	70%	50%	85%	70%	85%	50%	70%	50%	70%	50%	70%	50%	70%	50%	85%	50%	85%
Thiamine	85%	85%	50%	50%	50%	50%	50%	50%	50%	50%	50%	50%	50%	50%	70% i	50%	50%	50%	50%	50%
Riboflavin	95%	98%	50%	50%	50%	50%	95%	98%	50%	50%	70% i	50%	50%	50%	50%	50%	50%	50%	50%	50%
Niacin equiv	95%	95%	95%	98%	98%	98%	95%	95%	85%	85%	95%	98%	70%	85%	98%	98%	85%	85%	95%	95%
Vitamin C	85%	85%	98%	98%	98%	98%	95%	95%	93%	93%	85%	85%	95%	95%	95%	95%	93%	93%	70%	70%
Vitamin B6	95%	98%	70%	70%	70%	70%	50%	50%	70%	70%	50%	50%	50%	50%	50%	50%	50%	70%	50%	50%
Vitamin B12	50%	50%	50%	50%	50%	50%	50%	50%	50%	50%	50%	50%	50%	50%	50%	50%	50%	50%	50%	50%
Folate equiv	93%	98%	85%	93%	85%	85%	70%	85%	70%	70%	50%	50%	50%	70%	50%	50%	50%	50%	50%	50%
Vitamin A equiv	50%	50%	70%	70%	70%	70%	50%	50%	50%	50%	50%	50%	70%	70%	50%	50%	50%	50%	50%	50%
Magnesium	70%	98%	50%	70%	70% i	50%	50%	50%	85% i	50%	85% i	70% i	85% i	70% i	85% i	70% i	70% i	50%	85% i	70% i
Calcium	85%	95%	50%	50%	50%	70% i	50%	70%	70% i	85% i	85% i	95% i	85% i	93% i	85% i	95% i	70% i	70% i	70% i	85% i
Phosphorus	85%	93%	85%	85%	70%	85%	85%	95%	70%	70%	50%	70%	50%	50%	70%	70%	70%	85%	70%	85%
Iron	95%	98%	85%	85%	50%	50%	50%	50%	50%	50%	50%	50%	50%	50%	50%	50%	50%	50%	50%	50%
Zinc	50%	85%	50%	50%	70% i	50%	50%	50%	70% i	50%	70% i	50%	70% i	50%	70% i	50%	70% i	50%	70% i	50%
Selenium	>98%[a]	>98%[a]	70%	85%	95%	98%	50%	70%	93%	98%	70%	98%	98% i	85% i	70%	95%	70%	95%	50%	98%
Iodine	>98%[a]	>98%[a]	98% i	98% i	50%	50%	98%	98%	50%	50%	98% i	98% i	98% i	98% i	98% i	98% i	98% i	98% i	98% i	98% i

Note. M Male. F Female. [a] Exact probability unable to be calculated due to no daily variation in nutrient content. Red shading indicates high confidence (≥85%) of nutritional inadequacy, assuming long term usual intake of the diet. Yellow shading indicates moderate confidence (70%-85%) of nutritional inadequacy, assuming long term usual intake of the diet. Unshaded indicates ≥50% chance of nutritional adequacy, assuming long term usual intake of the diet.
