# Supplementary material for: Comparison of the Nutritional Adequacy of Current Food-Based Very Low Energy Diets: A Review and Nutritional Analysis
Source: Nutrients. 2024 Sep 5;16(17):2993. doi: 10.3390/nu16172993 (PMC11396843; doi:10.3390/nu16172993)
Supplement: Supplementary file 1 [file nutrients-16-02993-s001.zip › Poon_Nutrients Supp S5.pdf]

Table S5. Nutrient content of Optifast® and 9 food-based VLEDs as a proportion (%) of AI for adult males and females 19-50 years (Diet/AI x100).

Nutrient	Optifast®		Mosley 2015 [22]		Bailey 2016 [23]		Baldry 2017 [24]		Mosley 2019 [25]		Bailey 2019 [26]		Myers-Cooke 2020 [27]		Bailey 2021 [28]		Mosley 2021 [29]		Bailey 2022 [30]	
	M	F	M	F	M	F	M	F	M	F	M	F	M	F	M	F	M	F	M	F
Dietary fibre (g)	43%	52%	105 %	125 %	76%	91%	69%	83%	60%	72%	39%	47%	67%	80%	57%	68%	66%	79%	51%	61%
Vitamin E (mg)	208 %	297 %	206 %	295 %	186 %	266 %	62%	89%	141 %	201 %	130 %	186 %	101 %	145 %	143 %	204 %	126 %	181 %	127 %	182 %
Sodium (mg)	149-298 %	149-298 %	174-348 %	174-348 %	231-461 %	231-461 %	88-177 %	88-177 %	200-400 %	200-400 %	113-226 %	113-226 %	104-208 %	104-208 %	164-328 %	164-328 %	176-351 %	176-351 %	209-417 %	209-417 %
Potassium (mg)	71%	97%	84%	114 %	77%	104 %	82%	111 %	66%	90%	54%	74%	55%	75%	58%	78%	60%	82%	59%	80%
Linoleic acid	18%	28%	81%	131 %	51%	83%	19%	31%	48%	78%	40%	65%	27%	44%	41%	66%	62%	100 %	65%	106 %
alpha linolenic acid	47%	77%	225 %	366 %	63%	103 %	27%	44%	82%	134 %	66%	108 %	25%	40%	53%	87%	92%	150 %	84%	136 %

Note. Red shading indicates amounts <50% of AI. Yellow shading indicates amounts between 50% and 99% of AI. Unshaded cells indicate amounts ≥100% of AI.
