# Supplementary material for: Comparison of the Nutritional Adequacy of Current Food-Based Very Low Energy Diets: A Review and Nutritional Analysis
Source: Nutrients. 2024 Sep 5;16(17):2993. doi: 10.3390/nu16172993 (PMC11396843; doi:10.3390/nu16172993)
Supplement: Supplementary file 1 [file nutrients-16-02993-s001.zip › Poon_Nutrients Supp S6.pdf]

Table S6. Mean energy and nutrient provision of 9 food-based VLED as a proportion (%) of Optifast®.

Nutrient	Optifast®	Mosley 2015 (22)	Bailey 2016 (23)	Baldry 2017 (21)	Mosley 2019 (24)	Bailey 2019 (25)	Myers-Cooke 2020 (26)	Bailey 2021 (27)	Mosley 2021 (28)	Bailey 2022 (29)
Energy (MJ)	100%	158%	162%	169%	158%	123%	108%	135%	169%	185%
Energy (kcal)	100%	158%	160%	172%	157%	125%	109%	134%	169%	187%
Protein (g)	100%	103%	115%	128%	92%	102%	87%	105%	107%	115%
Total fat (g)	100%	327%	393%	160%	427%	300%	173%	307%	433%	567%
- Saturated (g)	100%	300%	400%	200%	500%	275%	175%	250%	500%	600%
- Polyunsaturated (g)	100%	1400%	800%	400%	800%	700%	400%	700%	1000%	1000%
- Monounsaturated (g)	100%	950%	1450%	450%	1550%	1150%	600%	1250%	1450%	2250%
Carbohydrate (g)	100%	89%	63%	207%	58%	44%	82%	56%	72%	40%
Dietary fibre (g)	100%	238%	177%	162%	138%	92%	154%	131%	154%	115%
Thiamine (mg)	100%	50%	50%	56%	50%	50%	50%	38%	50%	50%
Riboflavin (mg)	100%	39%	39%	100%	43%	30%	35%	35%	48%	43%
Niacin equiv (mg)	100%	111%	114%	111%	82%	107%	79%	118%	89%	96%
Vitamin C (mg)	100%	258%	268%	138%	148%	121%	138%	177%	145%	86%
Vitamin E (mg)	100%	100%	90%	29%	67%	62%	48%	67%	62%	62%
Vitamin B6 (mg)	100%	83%	62%	41%	59%	48%	34%	48%	55%	34%
Vitamin B12 (µg)	100%	68%	81%	130%	92%	57%	49%	70%	86%	76%
Folate equiv (µg)	100%	94%	93%	85%	73%	53%	68%	50%	54%	56%
Vitamin A equiv (µg)	100%	175%	135%	91%	77%	103%	127%	83%	69%	75%
Sodium (mg)	100%	117%	155%	59%	134%	76%	70%	110%	118%	140%
Potassium (mg)	100%	118%	107%	115%	93%	76%	77%	81%	84%	83%
Magnesium (mg)	100%	70%	58%	63%	48%	44%	43%	41%	50%	44%
Calcium (mg)	100%	60%	43%	79%	28%	20%	23%	21%	40%	29%
Phosphorus (mg)	100%	96%	86%	111%	78%	70%	63%	74%	83%	83%
Iron (mg)	100%	83%	43%	30%	43%	30%	39%	30%	35%	35%
Zinc (mg)	100%	62%	47%	56%	46%	37%	45%	45%	44%	53%
Selenium (µg)	100%	64%	68%	54%	59%	57%	33%	59%	58%	54%
Iodine (µg)	100%	22%	34%	61%	38%	18%	20%	19%	31%	20%
Linoleic acid(g)	100%	500%	350%	150%	300%	250%	200%	250%	400%	400%
alpha linolenic acid (g)	100%	483%	133%	50%	183%	150%	50%	117%	200%	183%

Note: Red shading indicates amounts <50% of Optifast®. Yellow shading indicates amounts between 50% and 99% of Optifast®. Unshaded cells indicate amounts ≥100% of Optifast®.
